# Church and Family Informal Social Support Networks of African Americans

**DOI:** 10.1093/geroni/igaa057.2270

**Published:** 2020-12-16

**Authors:** Robert Taylor, Linda Chatters

**Affiliations:** University of Michigan, Ann Arbor, Michigan, United States

## Abstract

Social support networks are an integral component of an individual’s life. This presentation investigates the complementary roles of family and church members as sources of informal social support among African Americans. The analysis utilizes the African American sub-sample of the National Survey of American Life. A pattern variable was constructed that describes four types of church and family networks: 1) received support from both family and church members, 2) received support from family members only, 3) received support from church members only, and 4) never received support from family nor church members. Overall the findings indicated 1) the majority of African Americans received support from both groups, 2) a small group of respondents were socially isolated in that they did not receive assistance from either family or church members, 3) for some African Americans who were estranged from their family members, church members were an alternative source of social support. Part of a symposium sponsored by the Religion, Spirituality and Aging Interest Group.

